# MicroRNA-34a inhibits human trophoblast cell invasion by targeting *MYC*

**DOI:** 10.1186/s12860-015-0068-2

**Published:** 2015-09-03

**Authors:** Manni Sun, Haiying Chen, Jing Liu, Chunxiao Tong, Tao Meng

**Affiliations:** Department of Obstetrics, The First Affiliated Hospital of China Medical University, 155 North Nanjing Street, Shenyang, 110001 People’s Republic of China

**Keywords:** microRNA-34a, *MYC*, Trophoblast invasion, Preeclampsia

## Abstract

**Background:**

Preeclampsia, one of the major disorders of pregnancy, is characterized by inadequate trophoblast invasion and defective trophoblast-mediated remodeling of placental vasculature. MicroRNA-34a (miR-34a) has been found to be aberrantly expressed in the placentas of preeclamptic patients, yet its role in placental development and in the pathogenesis of preeclampsia remains elusive.

**Results:**

The levels of miR-34a in the placentas of 20 preeclamptic patients and 20 healthy subjects were determined by real time-PCR, and miR-34a was found significantly elevated in the preeclamptic placentas. Further, the function of miR-34a in trophoblast cells was investigated by overexpressing miR-34a in JEG-3 trophoblast cell line. Overexpression of miR-34a in JEG-3 cells inhibited cell proliferation, migration and invasion. In addition, elevated expression of miR-34a reduced the expression of both endogenous and ectopic *MYC*. Moreover, we identified that *MYC* mRNA was a direct target of miR-34a in JEG-3 cells by dual luciferase reporter assay, and found that downregulation of *MYC* expression by miR-34a targeting significantly reduced the invasiveness of JEG-3 cells.

**Conclusions:**

Our findings provide preliminary evidence for the diverse functions of miR-34a in trophoblast biology, and suggest that miR-34a suppresses trophoblast invasion by directly targeting *MYC*.

## Background

Preeclampsia is one of the most frequently encountered medical complication of pregnancy and affects 3-5 % of pregnant women worldwide [1]. Preeclampsia is typically characterized by new-onset hypertension, proteinuria and other systemic disturbances in the second half of pregnancy, during labor, or in the early period after delivery [2], and it confers adverse pregnancy outcomes as well as maternal and fetal morbidity. The etiology of preeclampsia is not fully understood at present. Although enormous efforts has been invested in the recent decades in finding reliable biomarkers for early detection and management of preeclampsia, the results are disappointing due to a low detection rate or detection at later gestational ages [3]. Hence, understanding the molecular mechanisms underlying the pathogenesis of preeclampsia and searching for reliable early biomarkers are still the primary tasks for preeclampsia diagnosis and therapy.

Delivery of the placenta is the only known cure for preeclampsia, implying that placenta is the principle contributor to the pathogenesis of preeclampsia. During early normal placental development, extravillous trophoblasts of fetal origin invade the uterine spiral arteries of the deciduas and myometrium, and remodel the placental vasculature in order to allow sufficient placental perfusion to nourish the fetus. However, inadequate placental trophoblast invasion has been well documented in preeclampsia and it is generally believed to be the main cause for placental underperfusion and the development of preeclampsia [4, 1]. The molecular regulatory networks underlying trophoblast invasion have not been well defined. A number of genes that are associated with the invasiveness of carcinoma cells, such as matrix metalloproteinases and FOS transcription factors, have been demonstrated to play a role in trophoblast invasion *in vitro* [5, 6], yet a lot more remain to be explored.

MicroRNAs (miRNAs) are a class of endogenous small non-coding RNAs of about 22 nucleotides in size, and play pivotal roles in the post-transcriptional regulation of a variety of physiological activities by targeting messenger RNAs (mRNAs) for cleavage or translational repression [7]. miRNAs are transcribed by RNA polymerase II in the form of capped and polyadenylated primary transcripts (pri-miRNAs) which are cleaved by the Drosha ribonuclease III enzyme to produce approximately 70-nucleotide stem-loop precursor miRNAs (pre-miRNAs). Pre-miRNAs are further cleaved by cytoplasmic Dicer ribonuclease to generate mature miRNA and antisense miRNA products, and the mature miRNAs are incorporated into the RNA-induced silencing complexes that recognize the target mRNAs and interfere with their stability or translation [8]. Aberrant miRNA expression has been reported to be closely associated with multiple diseased conditions in human, including cancer [9], inflammation [10] and cardiovascular diseases [11]. Recently, miRNAs expression profiles in human placentas have been characterized, and the dynamically expressed miRNAs during pregnancy are thought to influence different aspects of trophoblast biology such as proliferation, syncytialization and invasion [12–14], whereas deregulated expression of miRNAs in trophoblast cells may lead to placental malfunction or placenta-related diseases such as preeclampsia [15, 16].

MiR-34a has been extensively studied in cancer in the aspects of tumor growth, invasion and metastasis [17, 18], yet little is known about the roles of miR-34a in trophoblast biology. Recently, Doridot and colleagues reported that pri-miRNA-34a was overexpressed, yet mature miR-34a was decreased, in the preeclamptic placentas [19]. Following the paradoxical findings, we first examined the levels of miR-34a in 20 preeclamptic placentas in comparison with 20 healthy placentas, and found that mature miR-34a was elevated in the preeclamptic placentas. Further, we investigated the functions of miR-34a in trophoblast proliferation, migration and invasion by overexpressing miR-34a in JEG-3 trophoblast cell line. In addition, we identified that *MYC* mRNA was a target of miR-34a in JEG-3 cells, and revealed that suppressing of *MYC* expression by miR-34a significantly inhibited JEG-3 invasion. These results suggest that miR-34a-mediated suppression of *MYC* expression may play a critical role in the regulation of trophoblast invasion and that excessive silencing may contribute to the pathogenesis of preeclampsia.

## Results

### Elevated level of miR-34a in preeclamptic placentas

**Fig. 1:**
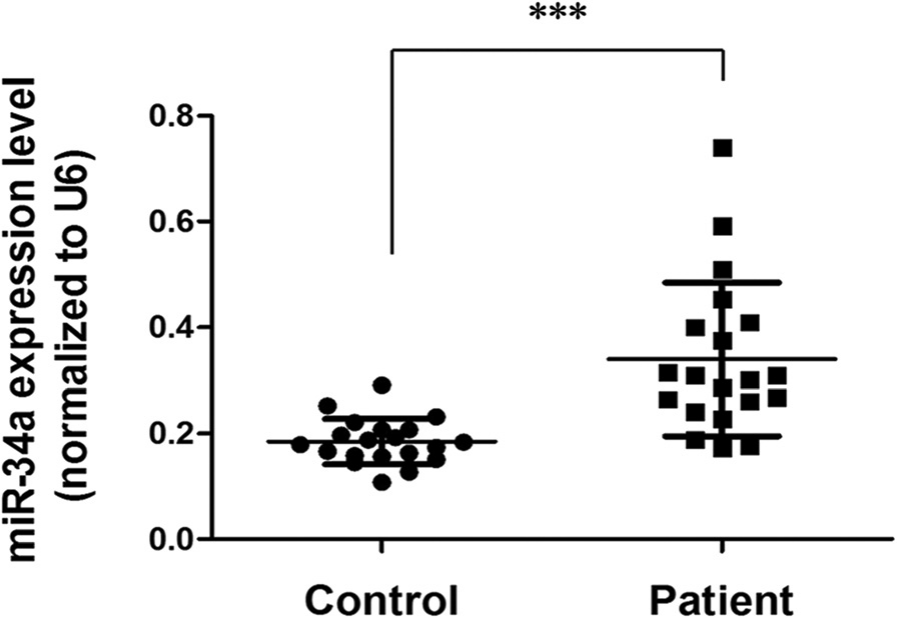
Expression of miR-34a in preeclamptic placentas and healthy placentas. Placentas of preeclamptic patients and healthy subjects were collected after delivery (*n* = 20 each). MiR-34a expression levels in the placental chorionic villi were determined by real-time PCR using U6 as the internal reference. ***p < 0.001

Twenty preeclamptic patients and 20 healthy control subjects were recruited in this study, and their clinical characteristics are summarized in Table 1. Compared to the healthy group, the preeclamptic patients exhibited elevated systolic and diastolic blood pressures and proteinuria, whereas the birth weight of the preeclampsia group was significantly lower. After delivery, the chorionic villi were isolated from the placentas, and the levels of miR-34a in the tissues were determined by real-time PCR using U6 as the internal control. As shown in Fig. 1, the levels of miR-34a in the preeclamptic placentas were significantly higher compared to the healthy placentas, implying that elevated of miR-34a may be involved in the pathogenesis of preeclampsia.

**Table 1:** Clinical characteristics of study subjects

Variables	Control (*n* = 20)	Preeclampsia (*n* = 20)	*P*-values
Maternal age (years)	28.9 ± 2.5	29 ± 3.7	0.921
Gestational age (weeks)	39 ± 2.6	38 ± 1.8	0.179
Systolic blood pressure (mm Hg)	118.9 ± 8.5	151.4 ± 12.5	<0.001
Diastolic blood pressure (mm Hg)	75.6 ± 8.2	94.7 ± 11.7	<0.001
Birth weight (g)	3554 ± 436	3135 ± 723	0.032
Mild proteinuria/Severe proteinuria^a^	15 %^b^/0	25 %/75 %	

^a^Mild proteinuria: 0.2-1 g/L protein in a random urine test; severe proteinuria: >1 g/L protein in a random urine test

^b^3 control subjects with mild proteinuria were not diagnosed to be PE because their systolic blood pressure and diastolic blood pressure were within the normal range

### Overexpression of miR-34a inhibited proliferation, migration and invasion of JEG-3 cells

**Fig. 2:**
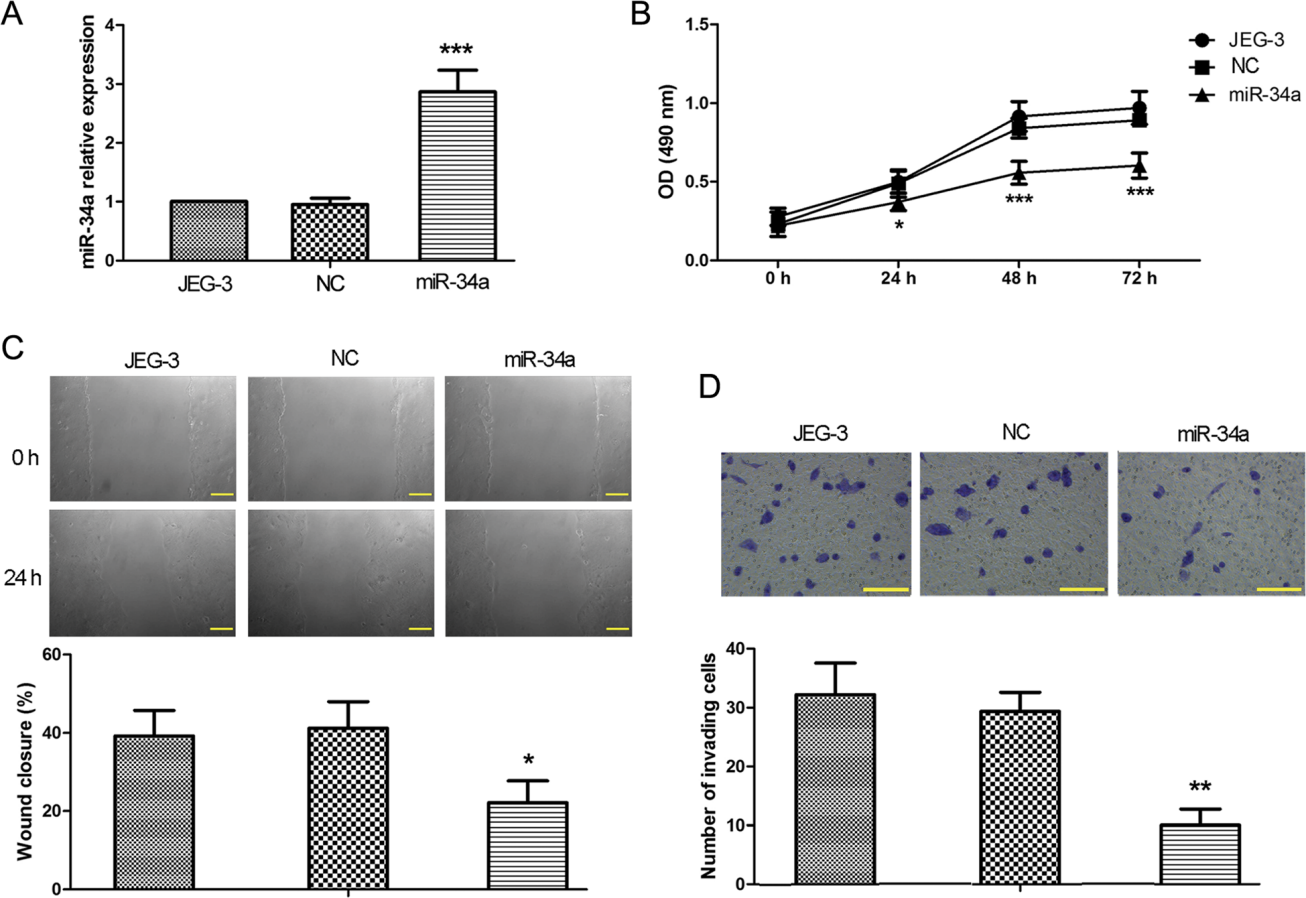
Overexpression of miR-34a inhibited proliferation, migration and invasion of JEG-3 cells. MiR-34a and non-targeting control (NC) sequence were transfected into JEG-3 cells. **a** The level of mature miR-34a was determined by real-time PCR analysis 24 h after transfection. **b** At 24 h after transfection, the cells were subjected to MTT assay to assess cell proliferation with 5 replicates for each time point. **c** Cell migration was measured by scratch wound assay. The cells were photographed at 100× magnification at 0 h and 24 h post-scratching, and the scale bars indicate 100 μm. **d** At 24 h after transfection, the cells were set up for *in vitro* Matrigel-based invasion assay to assess cell invasiveness. Following 24 h incubation, the invading cells on the bottom surface of the Transwell membrane were fixed, stained and photographed at 200× magnification (100 μm scales), and the average number of invading cells of 5 randomly selected fields from each sample were calculated. Each experiment was performed at least three times. The figure shows the representative images from repeated experiments and the values are expressed as the mean ± standard deviation. Compared with parental JEG-3 cells, *p < 0.05; **p < 0.01; ***p < 0.001

Human trophoblast cell line JEG-3 was transfected with miR-34a or non-targeting control (NC) expression vectors, and the level of miR-34a was remarkably elevated in the miR-34a-overexpressing cells compared with parental JEG-3 cells, whereas NC showed no effects on miR-34a expression (Fig. 2a). Overexpression of miR-34a in JEG-3 cells significantly reduced cell proliferation rate as assessed by MTT assay (Fig. 2b). Moreover, *in vitro* scratch assay revealed that elevated level of miR-34a slowed the migration rate of JEG-3 cells (Fig. 2c), and Matrigel-based invasion assay showed that the invasiveness of JEG-3 cells were prominently attenuated upon miR-34a overexpression (Fig. 2d). Taken together, these results indicated that overexpression of miR-34a significantly suppressed cell proliferation, migration and invasiveness of JEG-3 cells, suggesting that miR-34a may play critical roles in multiple aspects of trophoblast physiology.

### MiR-34a inhibited invasiveness of JEG-3 cells by targeting *MYC*

**Fig. 3:**
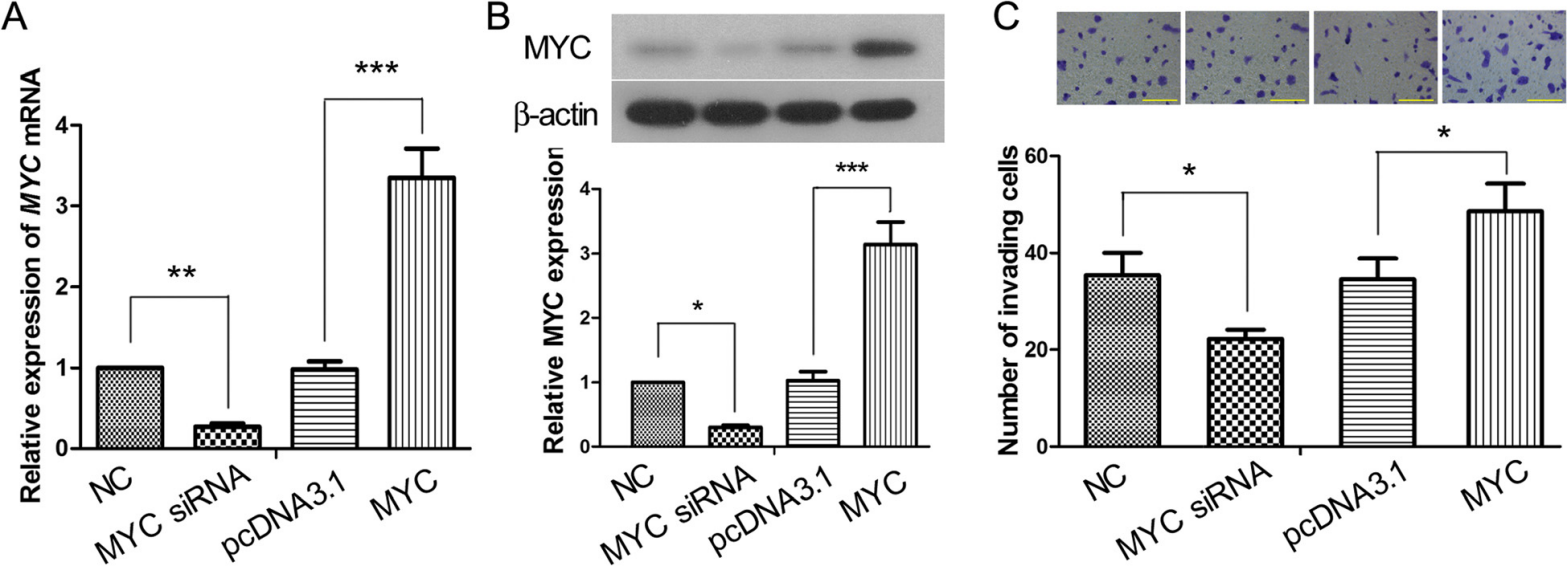
MYC regulates the invasion of JEG-3 cells. JEG-3 cells were transfected with MYC overexpression construct, MYC siRNA, or the corresponding empty vectors. 48 h later, the levels of **a** *MYC* mRNA and **b** MYC protein in the cells were analyzed by real-time PCR and Western blot analysis respectively, using β-actin as the internal control. **c** Matrigel-based Transwell invasion assay was set up 24 h after transfection, and the number of invading cells was counted 24 h later at 200× magnification (scale bars = 100 μm). This figure shows the representative images from three independent experiments, and the values are expressed as the mean ± standard deviation. *p < 0.05; **p < 0.01; ***p < 0.001

**Fig. 4:**
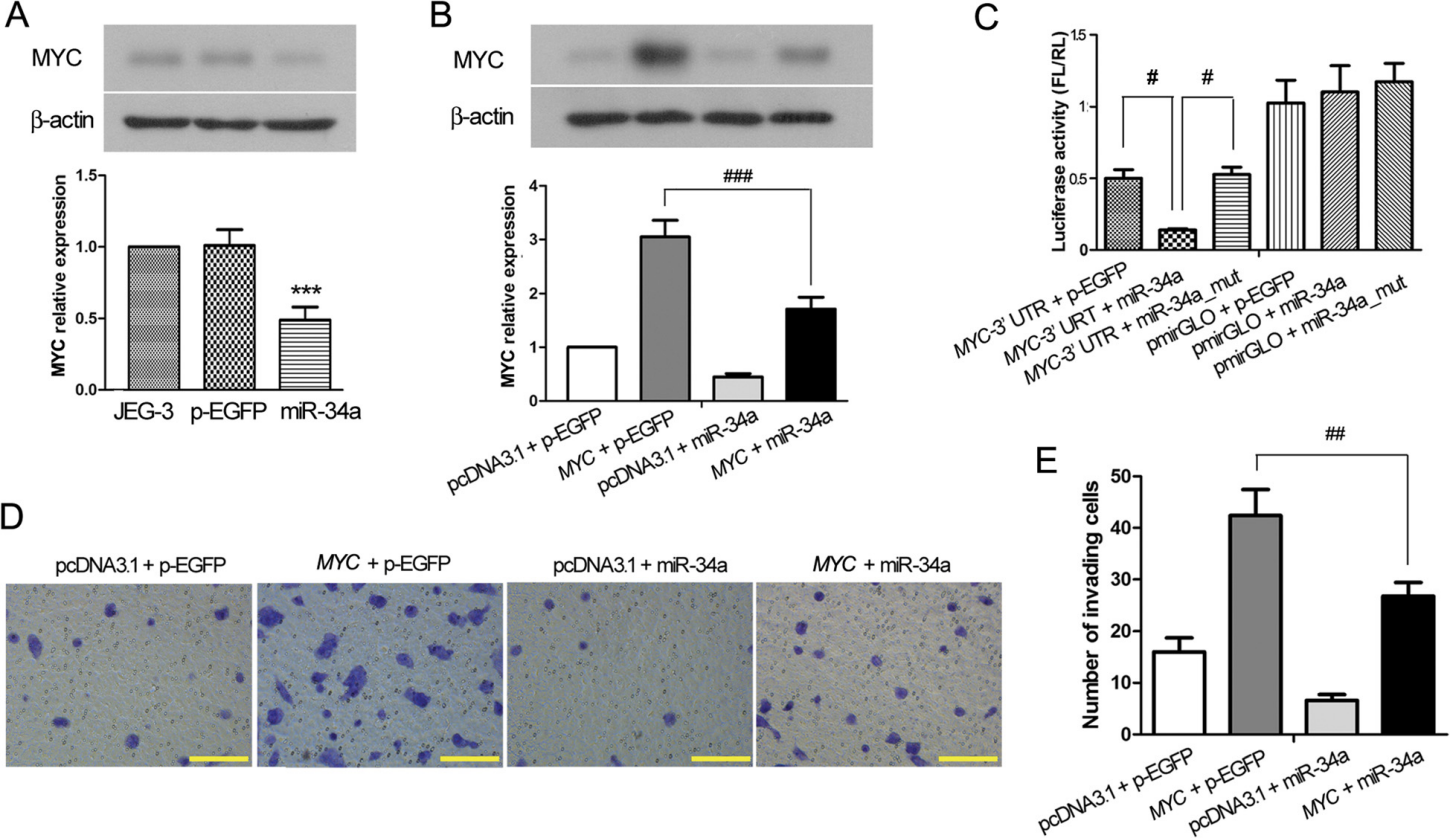
MiR-34a suppresses trophoblast invasion by targeting *MYC* mRNA. **a** Western blot was performed to assess the protein levels of MYC in non-transfected JEG-3 cells and in JEG-3 cells that were transfected with p-EGFP-miR-34a (miR-34a) or p-EGFP empty vector. β-actin was used as an internal control for grayscale analysis. Compared with the non-transfected cells, ***p < 0.001. **b** JEG-3 cells were co-transfected with pcDNA3.1-*MYC* (*MYC*) (or pcDNA3.1 vector) and p-EGFP-miR-34a (or p-EGFP vector), and the protein levels of MYC were examined by Western blot analysis 48 h after transfection. β-actin was used as an internal control, and grayscale analysis shows the mean ± standard deviation of three independent experiments (### *p* < 0.001). **c** *MYC* 3’-UTR was cloned into the firefly (FL) pmirGLO luciferase reporter vector. pmirGLO-*MYC* 3’-UTR (or pmirGLO vector) was co-transfected with p-EGFP vector, p-EGFP-miR-34a (miR-34a) or p-EGFP-miR-34a mutant (miR-34a_mut) into JEG-3 cells. Renilla (RL) luciferase reporter vector was also co-transfected as the internal reference. The luciferase activity (FL/RL) indicates the silencing effect resulting from the binding of miR-34a or miR-34a_mut to *MYC* 3’-UTR (# *p* < 0.05). **d, e** JEG-3 cells were co-transfected with *MYC* and miR-34a using the corresponding empty vectors as control, and the transfected cells were subjected to Matrigel-based invasion assay. The photographs of the invading cells were taken at 200× magnification, and the scales are 100 μm. The figure shows the representative images from three independent experiments and the values are expressed as the mean ± standard deviation. ## *p* < 0.01

We further investigated the underlying molecular mechanism for the inhibitory role of miR-34a in the invasiveness of JEG-3 cells. *MYC* is a well known oncogene and plays an important role in cancer cell migration and invasion [20]. However, whether MYC protein also plays a part in the invasion of trophoblast cells is unknown. By enhancing or silencing the expression of *MYC* in JEG-3 cells, we found that the invasiveness of JEG-3 cells was associated with the expression level of MYC (Fig. 3), indicating that MYC also plays an important role in regulating the invasion of JEG-3 cells. We then tested our hypothesis that miR-34a regulates the invasion of JEG-3 cell via targeting *MYC* mRNA. Western blot analysis showed that overexpression of miR-34a in JEG-3 cells downregulated the expression of endogenous MYC protein (Fig. 4a). JEG-3 cells were further co-transfected with *MYC* and miR-34a expression vectors, using the corresponding empty vectors pcDNA3.1 and p-EGFP as the controls. As shown in Fig. 4b, the level of MYC protein was markedly elevated in the cells transfected with *MYC* expression vector, and the level was significantly reduced by co-expression of miR-34a, suggesting that miR-34a regulates the expression *MYC* in JEG-3 cells. Furthermore, direct binding of miR-34a on the 3’-untranslated region (UTR) of *MYC* mRNA was assessed by dual luciferase reporter assay. *MYC* 3’-UTR sequence containing the putative miR-34a binding site was PCR-amplified and cloned into a firefly luciferase reporter vector pmirGLO. In JEG-3 cells, the firefly vectors with and without insertion of *MYC* 3’-URT were co-transfected with miR-34a expression vector, mutant miR-34a (miR-34a_mut) expression vector or p-EGFP empty vector. At 48 h after transfection, luciferase activity was significantly reduced in the MYC reporter cells overexpressing miR-34a, whereas mutation in miR-34a abolished the translational inhibition (Fig. 4c). Hence, the luciferase assay demonstrated that miR-34a bound directly and specifically to *MYC* 3’-URT and repressed the translation of *MYC* in JEG-3 cells. We further assessed the effect of miR-34a-mediated downregulation of MYC on the invasiveness of trophoblast cells. Matrigel-based invasion assay showed that overexpression of MYC protein enhanced the invasiveness of JEG-3 cells, and such MYC-enhanced invasion was significantly suppressed by co-expression of miR-34a (Fig. 4d, e). In summary, the above results suggest that MYC promotes trophoblast invasion, while miR-34a inhibits trophoblast invasion by downregulating MYC expression via direct targeting on *MYC* transcripts.

## Discussion

Trophoblast cells are a heterogeneous group of cells forming the fetal-maternal interface and exhibiting a wide spectrum of functions. First trimester and term trophoblast cells display different miRNA fingerprints, implying that dynamic expression of miRNAs may play critical roles in the regulation of differential behaviors of trophoblast cells at various stages during placentation [12], whereas deregulated miRNAs during this physiological process can lead to placental malfunction and related diseases such as preeclampsia [16, 15]. Doridot et al. reported that pri-miR-34a was overexpressed in the preeclamptic placentas but the mature miR-34a level was decreased [19]. In this study, we first examined the level of mature miR-34a in preeclamptic placentas, and found that the mature miR-34a was indeed elevated in the preeclamptic placentas, which is consistent with the pattern of pri-miR-34a in Doridot’s work. Doridot et al. extended the miRNAs by the non-specific poly-adenylation method before real-time PCR analysis, and here, we used a stemloop primer-based method, which has been demonstrated to be more efficient and specific [21]. Hence, we believe that the contradictory results of pir-miR-34a and mature miR-34a in Doridot’s work may be attributed to the technical flaw in miRNA extension.

The role of miR-34a in placental development and in the pathophysiology of preeclampsia is unclear. In the present study, using JEG-3 trophoblast cell line as the model, we demonstrated that overexpression of miR-34a inhibited cell proliferation, migration and invasion, suggesting that miR-34a plays important roles in many aspects of trophoblast biology. Further investigation on the molecular target of miR-34a revealed that miR-34a directly bound to the 3’-UTR of *MYC* transcripts and suppressed *MYC* expression. Moreover, the invasiveness of JEG-3 cells were largely dependent on the level of MYC protein, which was regulated by miR-34a, suggesting that targeting *MYC* by miR-34a may play a critical role in the regulation of trophoblast invasion, and that excessive suppression of *MYC* translation by miR-34a may result in inadequate trophoblast invasion and the development of preeclampsia.

Inadequate trophoblast invasion is so far recognized as a major pathological factor for preeclampsia [1, 4]. Extensive work has been conducted to address the molecular mechanisms underlying the impaired trophoblast invasion, and a number of cancer-related molecules have been identified to play a role in trophoblast invasion [6, 5, 22]. Because of the inherently invasive nature, trophoblast cells are often compared with highly invasive carcinoma cells, and in many cases, both exhibit similar gene expression signatures that regulate cell invasion. Our study demonstrated that the levels of miR-34a and MYC, which have been shown to regulate tumor cell invasion and metastasis [20, 23], significantly affected the invasive capacity of JEG-3 cells. These observations are consistent with their functions in cancer. Although the functions of miR-34a or MYC in placental development have been rarely reported, numerous studies on miR-34a and MYC have been done in cancer. MiR-34a was reported to directly target *MYC* mRNA in lymphoma and interfere with p53-dependent apoptotic pathway [24]. In addition, miR-34a can indirectly regulate MYC expression through Wnt signaling pathway in glioma [25]. Here, we demonstrated that miR-34a could directly targeted *MYC* mRNA and modulate its expression in trophoblast cells, thereby regulating the invasiveness of the cells. Interestingly, previous studies have shown that the transcription of *MIR34A* is repressed by MYC protein via binding in the vicinity of the *MIR34A* gene promoter [26], suggesting that a regulatory feedback loop may exist between miR-34a and MYC, and disruption of the balance between these two molecules can result in cell dysfunction, and in this case, impaired trophoblast invasion.

Although JEG-3 is widely used in the studies on trophoblast biology, researchers should bear in mind that JEG-3 is derived from choriocarcinoma, thus it inherits typical properties of tumor cells, such as accelerated proliferation and enhanced invasiveness. Hence, the functions of miR-34a in trophoblasts should be verified in primary trophoblast cells. In our preliminary experiments, we indeed observed that overexpression of miR-34a suppressed proliferation and invasiveness of placental cells from crude collegenase digestion of chorionic villi (data not shown). In future studies, the role of miR-34a in trophoblast proliferation should be assessed in primary villous trophoblasts, while its role in trophoblast invasion should be done using primary extra villous trophoblasts, wherein high purity of the isolated cells is the key.

Preeclampsia may cause maternal and fetal morbidity and mortality, and requires close medical monitoring. In the recent decades, numerous effects have been invested to find an efficient approach for the early detection of preeclampsia. Serum levels of the soluble proteins that are involved in placental angiogenesis, such as vascular endothelial growth factor receptor-1, placental growth factor and endoglin, have been proposed as potential markers, but the results are unsatisfactory [3]. Recently, studies have shown that fetal cell-free nucleic acids can enter maternal circulation and may reflect the pathological conditions in placental diseases like preeclampsia [27]. Hence, it is intriguing and clinically significant to know whether the level of miR-34a in the maternal plasma circulation could serve as a potential biomarker for preeclampsia, which is to be addressed in future studies.

## Conclusions

In this study, we reported elevated levels of miR-34a in the preeclamptic placentas and demonstrated that miR-34a can suppress proliferation, migration and invasion of JEG-3 trophoblast cells. Moreover, miR-34a may regulate trophoblast invasion by targeting *MYC*. Our findings suggest that miR-34a plays a critical role in trophoblast biology and it may be also involved in the pathophysiology of preeclampsia.

## Methods

### Tissue collection

Ethnic approval for the collection and experimentation of human placental tissues was obtained from the Clinical Research Ethics Committee of China Medical University, and all procedures in this study were carried out in strict accordance with the approved protocol*.* Written informed consents were obtained from all participants. Preeclamptic placentas and healthy placentas (*n* = 20 each) were obtained after delivery from preeclamptic patients and healthy subjects who were recruited at The First Affiliated Hospital of China Medical University. Fragments from the placental subchorial zone were dissected. Maternal membranes were eliminated, and floating villi were washed in PBS (pH 7.2) and subjected to RNA extraction.

### Real-time PCR

Total RNA was extracted with the RNAsimple Total RNA Extraction Kit (TIANGEN Biotech, Beijing, China) following the manufacturer’s protocol. Reverse transcription of mature miR-34a was conducted using a stemloop primer-based method as previously described [21]. Quantitative real-time PCR was performed using SYBR GREEN PCR Master Mix (Solarbio, Beijing, China), and fluorescence amplification was detected by an Exicycler 96 Real-Time Quantitative Thermal Block (Bioneer, Daejeon, Korea). The PCR primers for miR-34a were designed as one strand complementary to miR-34a (5′-GGCGATGGCAGTGTCTTAGC-3′) and the other strand to the stemloop RT primer (5′-GTGCAGGGTCCGAGGTATTC-3′); U6 was used as the internal control for miRNAs with the following primers: U6 forward, 5′-CTCGCTTCGGCAGCACA-3′, and U6 reverse, 5′-AACGCTTCACGAATTTGCGT-3′. The PCR primers for *MYC* mRNA were: *MYC* forward, 5′-CAGCGACTCTGAGGAGGAACA-3′, and *MYC* reverse, 5′-GCTGCGTAGTTGTGCTGATGTG-3′. *ACTB* mRNA (encoding β-actin) was used as the internal control for mRNAs, and the primers were: *ACTB* forward, 5′-CTTAGTTGCGTTACACCCTTTCTTG-3′, and *ACTB* reverse, 5′-CTGTCACCTTCACCGTTCCAGTTT-3′.

### Cell culture

JEG-3 trophoblast cell line which was initially derived from metastatic lesions of choriocarcinoma was purchased from the Cell Bank of Chinese Academy of Sciences (Shanghai, China). JEG-3 cells were cultured in DMEM (Gibco, Carlsbad, CA, USA) supplemented with 10 % FBS (FBS, Hyclone, Logan, UT, USA) at 37 °C in an atmosphere of 5 % CO_2_ for no more than 20 successive passages.

### Vector construction and Transfection

MiR-34a expression construct was established by PCR amplification of pri-miR-34a from JEG-3 genomic DNA with the following primer set: forward 5′-AATGGATCCTAGTTGCCTGGGCTGGTCTT-3′ (with BamHI restriction site) and reverse 5′- GTCGAATTCCGCTTCATCTTCCCTCTTGG-3′ (with EcoRI restriction site). The PCR-amplified fragments were then inserted into p-EGFP-N1 vectors (TaKaRa Clontech, Otsu, Japan) to make p-EGFP-miR-34a. The coding sequence of *MYC* was amplified by PCR from JEG-3 cDNA library with the primers as follows: forward 5′-AGTGGATCCTCGCTGGATTTTTTTCGGGTAG-3′ (with BamHI restriction site); reverse 5′-TCCGAATTCCCTTACGCACAAGAGTTCCG-3′ (with EcoRI restriction site). PCR-amplified *MYC*-coding sequences were cloned into pcDNA3.1 vectors (TaKaRa Clontech) to generate pcDNA3.1-*MYC*. The oligonucleotides containing a short interfering RNA (siRNA) targeting *MYC* mRNA, (5′-GATCCCCGAGGGTCAAGTTGGACAGTTTCAAGAGAACTGTCCAACTTGACCCTCTTTTT-3′, sense strand, with BamHI/HindIII restriction sites), or a non-targeting control (NC) sequence (5′-GATCCCCTTCTCCGAACGTGTCACGTTTCAAGAGAACGTGACACGTTCGGAGAATTTTT-3′, sense strand, with BamHI/HindIII restriction sites) were cloned into the pRNA-H1.1/Neo siRNA expression vector (GenScript, Piscataway, NJ, USA), and the constructs were named *MYC* siRNA and NC respectively.

For the single-transfection experiments, JEG-3 cells were transfected with the indicated vectors using lipofectamin 2000 (Invitrogen, Carlsbad, CA, USA) according to the manufacturer’s instructions. The cells were starved in serum-free medium for 1 h prior to transfection and the medium was replaced with fresh culture medium containing 10 % FBS 6 h after transfection. For the double-transfection experiments, p-EGFP or miR-34a were transfected into JEG-3 cells 12 h after the transfection with pcDNA3.1 or *MYC* in the same method as single-transfection.

### Cell proliferation/MTT assay

Cell proliferation was assessed by 3-(4,5-dimethylthiazol-2-yl)-2,5-diphenyltetrazolium bromide (MTT) assay. JEG-3 cells were seeded at a density of 2 × 10^3^ cells/well in a 96-well plate and allowed to adhere before transfection. At 24 h after transfection, the medium was changed to fresh culture medium (0 h) and MTT (Sigma-Aldrich) was added to the medium at 24 h, 48 h and 72 h to a final concentration of 0.2 mg/ml, followed by 4 h incubation at 37 °C. Thereafter, the supernatant was carefully aspirated, and 200 μL DMSO (Sigma-Aldrich) was added to each well. The crystals were fully dissolved by vigorous shaking for 15 min at 37 °C in the dark. Optical density (OD) values at 490 nm were measured with the microplate reader ELX-800 (BioTek, Winooski, VT, USA), and the growth curves were plotted with the mean values of 5 replicates.

### Scratch wound assay

Scratch wound assay is a well-developed method to measure cell migration *in vitro* [28]. At 24 h post-transfection, a confluent monolayer of JEG-3 cells were incubated with 5 μM mitomycin-C (Sigma-aldrich) for 2 h to inhibit cell proliferation. The cells were washed, and a scratch to the cell monolayer was evenly created by horizontally dragging a 200 μl pipette tip across the surface of the monolayer. The cells were washed twice with serum-free medium to remove detached cells, and cultured with serum-free medium for 24 h at 37 °C in a humidified environment consisting of 5 % CO_2_. Cells were photographed under an inverted microscope at 0 h and 24 h post-scratching, and the percentage of wound closure was calculated as (original gap distance - gap distance at 24 h)/original gap distance × 100 %.

### Transwell assay

Transwell assay was performed to assess cell invasion *in vitro* 24 h after transfection. JEG-3 cells were pre-treated with 5 μM mitomycin-C for 2 h to inhibit proliferation. For each Transwell assay, 5 × 10^4^ cells resuspended in 200 μl serum-free medium were plated in the upper chamber of Transwell apparatus (Corning, New York, USA) that was pre-coated with phenol red-free Matrigel (BD Biosciences, San Jose, CA, USA), and the Transwell chambers were placed in 24-well plates with 800 μl culture medium (containing 20 % FBS) in each well. Cells were cultured for 24 h at 37 °C. Thereafter, the cells and Matrigel on the top surface of the Transwell microporous membrane were wiped off with a cotton swab. The cells on the bottom of the membrane were fixed with paraformaldehyde and stained with hematoxylin (Solarbio). Under a 200× inverted microscope, five random fields on each membrane were selected, and the average numbers of invading cells were determined.

### Western blotting

JEG-3 cells were transfected with the indicated vector(s) in the method described above. At 48 h post-transfection, protein levels of MYC in the transfected cells were determined by Western blot analysis. Cells were lysed with NP-40 lysis buffer (Beyotime, Beijing, China), and total proteins were extracted following the standard protocol. A total of 40 μg proteins from each sample were separated by SDS-PAGE and transferred to a PVDF membrane (Millipore, Bedford, MA, USA). The membrane was blocked with 5 % non-fat milk solution, and incubated with rabbit anti-human MYC primary antibody (1:1000; Santa Cruz, Dallas, TX, USA) overnight at 4 °C. Subsequently, the membrane was incubated with horseradish peroxidase (HRP)-labeled goat anti-rabbit IgG secondary antibody (1:5000; Beyotime) at room temperature for 45 min, followed by signal detection with the ECL solution (7SeaPharmTech, Shanghai, China). To verify equal loading and transfer, the membrane was stripped with the stripping buffer (Beyotime) and re-probed with anti-β-actin antibody (Santa Cruz). Densitometric analysis of the films was performed with Gel Pro Analyzer software using β-actin as the internal reference.

### Dual luciferase reporter assay

Direct binding of miR-34a to *MYC* 3’-UTR was assessed by dual luciferase reporter assay. The 3’-UTR of *MYC* mRNA that contains the putative miR-34a binding site was PCR-amplified from JEG-3 cDNA library with the following primers: forward 5′-ATGCTAGCTGCTACGGAACTCTTGTGCG-3′ (with NheI restriction site) and reverse 5′-GGCTGTCGACCTGTGTAACTGCTATAAACG-3′ (with SalI restriction site), and inserted into Firefly (FL) pmirGLO dual-luciferase miRNA target expression vector (Promega, Waltham, MA, USA). MiR-34a mutant was constructed by site-directed mutagenesis through PCR amplification using p-EGFP-miR34a as the template as well as the mutagenic primer 5′-GTTTCTTTACAGTCGTCTTAGCTG-3′ and its complimentary sequence. The PCR product was named p-EGFP-miR-34a_mut. JEG-3 cells were transfected with either pmirGLO-*MYC*-3’-UTR or pmirGLO vector, in combination with p-EGFP-miR34a, p-EGFP-miR34a_mut or p-EGFP vector, using the method described earlier. Renilla (RL) luciferase reporter vector (pRL-TK) (Promega) was co-transfected as the internal reference. At 48 h post-transfection, cell lysates were collected, and luciferase activity was measured with the dual-luciferase reporter assay system (Promega) according to the manufacturer’s instructions. Binding affinity of miR-34a or miR-34a_mut to *MYC* 3’-UTR was evaluated by the ratio of FL/RL activity.

### Statistical analysis

All experiments were replicated for a minimum of three times. Data are presented as the mean ± standard deviation. The levels of miR-34a in the placentas of preeclamptic patients and healthy subjects were compared with unpaired Student’s *t*-test. For *in vitro* experiments, one way analysis of variance (ANOVA) followed by Bonferroni *post-hoc* test was used to analyze differences between multiple groups. Raw data were analyzed with GraphPad PRISM software (version 5.0; San Diego, CA, USA). Regardless of statistical test used, the difference was considered statistically significant when *p* ≤ 0.05.
